# Assisted Suicide for Irreversible Patients on Life Support? The Intricate Italian Journey Towards Conforming with the Legislation of Spain, Austria, and Portugal

**DOI:** 10.3390/healthcare13080885

**Published:** 2025-04-11

**Authors:** Gianluca Montanari Vergallo, Susanna Marinelli, Nicola Di Fazio, Simona Zaami, Paola Frati

**Affiliations:** 1 Department of Anatomical, Histological, Forensic and Orthopedic Sciences, Sapienza University of Rome, 00161 Rome, Italy; gianluca.montanarivergallo@uniroma1.it (G.M.V.); paola.frati@uniroma1.it (P.F.); 2 School of Law, Polytechnic University of Marche, 60121 Ancona, Italy; s.marinelli@pm.univpm.it; 3 Department of Life Sciences, Health and Health Professions, Link Campus University, 00165 Rome, Italy; n.difazio@unilink.it

**Keywords:** right to die, assisted suicide, self-determination, dignity, palliative care, Constitutional Court comparative law, end-of-life decisions

## Abstract

**Background/Objectives**: In 2019, the Italian Constitutional Court (ICC) stated that the principles of equality, dignity, and self-determination enshrined in the constitution require that assisted suicide be considered lawful under certain conditions, including that the patient is kept alive through life-sustaining treatments. In fact, since such patients could already die by refusing treatment, assisted suicide is ethical as it allows them to die more quickly and with dignity. The paper aims to analyze the requirement of life-sustaining treatments from a legal and comparative perspective. **Methods**: The authors performed the search on Italian legal databases as well as on Scopus and PubMed and by comparing Italian regulations with those of Spain, Portugal, and Austria, which are similar to the Italian one in their fundamentally restrictive nature. The authors have delved into the Italian legal system through an analytical method of interpretation of the normative texts and used the comparative method to investigate which of the legal systems considered is more permissive. **Results**: According to the ICC, continuing to prohibit assisted suicide for patients who do not require life-sustaining treatments is not discriminatory: these patients cannot be equated with others, as only in the former case does refusing treatment lead to death. From its personalist ethical framework, the ICC also rejected the claim that the patient’s self-determination is being infringed upon: self-determination must be balanced with the protection of life, which is a fundamental value. However, in 2024, the ICC clarified that life-sustaining treatments are not limited to those directly supporting vital functions through medical machines, but also include all treatments without which the person would die in a short time, such as manual bowel evacuation. **Conclusions**: The current Italian regulation seems inconsistent. It would be preferable to regulate assisted suicide by referencing the models of Spain, Portugal, and Austria.

## 1. Introduction

The “right to die” is slowly and painstakingly establishing itself worldwide, in places as diverse as Benelux countries [1,2], Colombia [3], Canada [4], Ecuador [5], Spain [6], France [7], Portugal [8], Germany [9], and Austria [10,11]. In other countries, however, including Italy, in the absence of legislative measures, the essential duty to meet the needs and demands for protection from civil society has been entrusted to the judiciary [11,12].

In the Italian legal system, end-of-life issues are regulated by the Constitutional Court, which, in its 242/2019 ruling [13] concerning the case of Fabiano Antoniani [14], identified four criteria for admitting a patient to assisted suicide: (a) the patient must be suffering from an irreversible condition; (b) the condition must cause unbearable physical or psychological suffering; (c) the patient must be kept alive through life-sustaining treatments; and (d) the patient must be capable of making free and informed decisions. In addition to these substantive requirements, the court added two procedural requirements: (1) the procedures for assisted suicide must be carried out “in the manner prescribed by Articles 1 and 2” of Law No. 219/2017 [15]; (2) a public healthcare facility must verify whether the above conditions are met, subject to the opinion of the local ethics committee [16] as per regulations on informed consent and advance medical directives [17,18,19,20,21].

In light of the abovementioned court ruling, in Italy causing death is still a crime even if the patient asks for the lethal injection (euthanasia); it is only allowed to help the patient to commit suicide, possibly by biting a device that injects a lethal substance (assisted suicide). This ruling also establishes that assisted suicide must take place in public facilities, but do not indicate a specific category of doctors. Therefore, all doctors can provide this help, though they are not bound to perform it: the Constitutional Court recognizes conscientious refusal as a right of all health professionals [13].

The requirements and procedural conditions for assisted suicide established by Judgment no. 242/2019 [13], including the necessity for life-sustaining treatments, were confirmed by the Italian Constitutional Court in 2024 [22]. The case revolved around a criminal trial concerning three individuals accused of aiding a patient afflicted with multiple sclerosis in traveling to a Swiss clinic to seek assisted suicide. The patient had been enduring increasingly severe suffering, both physically and psychologically, due to disease progression. Consequently, he consistently expressed his voluntary and well-informed desire to pursue assisted suicide. Despite his need for assistance in basic daily tasks due to the progressive paralysis of his limbs, no medical professional deemed it necessary to provide life-sustaining interventions, as the patient did not rely on life-saving medications, artificial nutrition, hydration, or ventilation (Order no. 207/2018 and Judgment no. 242/2019).

The three accused individuals escorted him to the Dignitas clinic in Switzerland in order for him to receive assistance in dying, as was his wish. Their fundamental goal was to raise awareness among the general public and in the judiciary as to the pressing need to ease the strict criteria for accessing assisted suicide, specifically by eliminating the requirement for life-sustaining interventions to remove an unjust discriminatory element among patients. Upon their return to Italy, the trio voluntarily reported themselves to the carabinieri. The presiding judge at the Florence Court analyzed the case and concluded that only three out of the four conditions stipulated in Judgment no. 242/2019 were satisfied, as the patient was not reliant on life-sustaining treatments. In Order no. 32 dated 17 January 2024, before evaluating whether or not to convict the three defendants, the Court of Florence asked the Constitutional Court to eliminate the stipulation of dependence on life-sustaining interventions, deeming it incompatible with constitutional principles of equity, therapeutic autonomy, human dignity, and the right to privacy as enshrined in the European Convention on Human Rights [23].

The Florence Court raised constitutional legitimacy concerns regarding Article 3, arguing that making the conduct of those assisting a patient who depends on life-sustaining treatments to die lawful creates, in fact, “an unreasonable disparity of treatment compared to all other patients who live with intolerable subjective suffering due to irreversible conditions,” but are not connected to such medical devices [23].

The Constitutional Court rejected this claim, pointing out that Judgment no. 242/19 did not recognize a general right to end one’s life in every situation of intolerable suffering, whether physical or psychological, resulting from an irreversible condition [22,24]. According to the Constitutional Court, assisted suicide was permitted only for patients undergoing, or about to undergo, life-sustaining treatments, as only these patients are in conditions so severe that they can die by refusing such treatments, a right recognized by Law no. 219/2017 in accordance with Article 32, second paragraph, of the constitution. It would be unreasonable to allow them to die by refusing care, thereby exposing them to a phase of agony, yet deny them the right to die through assisted suicide, which would spare both the patients and their families much physical and psychological suffering. In other words, the interruption of life-sustaining treatment would cause physical suffering. The palliative treatments necessary to eliminate this physical suffering would be a source of loss of dignity for the patient and of internal suffering for them and their loved ones throughout the time separating said patients from death. Consequently, assisted suicide cannot be performed on patients who are not dependent on life-sustaining treatments, as they do not (or not yet) have the option to die simply by refusing care. Therefore, the two situations are different, and there is no unreasonable disparity of treatment [22].

As for therapeutic self-determination, according to the Florence Court, the requirement of dependence on life-sustaining treatments conflicts with Articles 2, 13, and 32, second paragraph, of the constitution, as it limits the patient’s freedom of therapeutic self-determination in choosing therapies, including those aimed at relieving suffering, without being justified by counter-interests of similar importance [23].

The Constitutional Court also found this objection to be unfounded. The Constitutional Court reiterated its previous decision [25], affirming that legalizing assisted suicide or euthanasia practices, on the one hand, increases a person’s autonomy in deciding their own fate, but on the other hand, creates risks that the legal system must prevent, fulfilling its duty to protect human life, which also arises from Article 2 of the constitution. These risks also include “the possibility that, in the presence of permissive legislation not accompanied by the necessary substantial and procedural safeguards, indirect ’social pressure’ could be created on other ill or simply elderly and lonely individuals, who might come to see themselves as a burden to their families and society as a whole, and thus decide to end their lives prematurely.” It is precisely this duty of the state to protect the life of every individual, as outlined in Article 2 of the constitution, that justifies the existence of requirements and limits to access assisted suicide [22].

The Florence Court also argues that the requirement of dependence on life-sustaining treatments violates the “principle of human dignity” because it forces a terminally ill patient, suffering irreversibly and intolerably, to wait until the worsening of their condition necessitates life-sustaining treatments, enduring the additional painful consequences of this situation. Therefore, to end their life, the patient must undergo a slower, more painful process, which is less in line with their vision of dignity in dying [23].

The Constitutional Court clarified that the concept of dignity is both objective and subjective in nature. Considering life-sustaining treatments necessary does not violate dignity in the objective sense, as there are no lives that are objectively unworthy of being lived; every life carries an inalienable dignity, regardless of the conditions in which it unfolds [22,26,27,28]. Objective dignity must necessarily be respected, even when the person themselves consents to the violation of their dignity. A typical example is the game of “dwarf tossing” [29] which was banned because it conflicted with the basic notion of respect for human dignity, even though those involved were consenting adults.

According to the Constitutional Court, the subjective notion of dignity coincides with self-determination, which is based on the ability of every individual to make fundamental life-or-death decisions (Articles 2, 13, and 32 of the constitution). Consequently, like self-determination, patient dignity must be weighed against the need to protect vulnerable individuals from decisions that may end their lives and are not entirely free. Therefore, the patient’s self-determination must in turn be reconciled with the duty to protect human life, and the legislator has a broad margin of discretion in finding the most appropriate balance [22,30,31,32]. Thus, the requirement for life-sustaining treatments does not violate dignity, even in the subjective sense [22,33,34,35].

Finally, the Florence Court argues that the prohibition of assisted suicide for patients who are not dependent on life-sustaining treatments, but who suffer from irreversible diseases and are capable of making decisions, violates the right to private life (enshrined in Article 8 of the ECHR) and creates discrimination (Article 14 of the ECHR). In relation to Article 8, it is argued that it would imply interference with the right to respect for private and family life, which is neither functional nor necessary for the protection of the right to life. Moreover, allowing assisted suicide only for some terminally ill patients—those specifically undergoing life-sustaining treatments—would be discriminatory and thus in violation of Article 14 of the ECHR [23].

The Constitutional Court has deemed such an objection ill founded. Regarding Article 8, the European Court of Human Rights (ECtHR) has affirmed that the right to decide by what means and when one’s life should end is an aspect of respect for private life [22,36,37,38]. However, it has also stated that states have considerable discretion in balancing this right with reasons for protecting human life, and concluded that it is up to individual states to assess the social implications and the risks of abuse and error that any legalization of medically assisted suicide inevitably entails. The Constitutional Court further excluded the possibility of discrimination, and thus a violation of the equality principle under Article 14 of the ECHR, between those who refuse life-sustaining treatments and those who, despite wishing to die due to their suffering, are not receiving such treatments and thus cannot refuse them to end their lives. According to the Constitutional Court, the two situations are not comparable, as demonstrated by the Oviedo Convention [22] recognizing the right to refuse medical interventions, while not safeguarding any interest related to medically assisted suicide.

The Constitutional Court concludes its decision by reiterating that the state has a duty, under Articles 2, 3, second paragraph, and 32 of the Italian Constitution, as well as Article 2 of the ECHR, to ensure that these patients receive all appropriate therapies, including palliative care, necessary to eliminate or at least reduce the suffering caused by their illness, and to provide them with any necessary support, whether medical, social, economic, or psychological [39]. It thus urges the legislature to strengthen and make effective palliative care (Law no. 38/2010) [22,40], which is distinct from deep continuous sedation [37,38,39]. These services are provided in hospitals, but also at home, thus supporting both families and healthcare professionals during the patient’s illness and after death [40]. It is likely for these reasons that where palliative care is implemented, the use of assisted suicide or euthanasia drastically decreases [41,42,43,44].

The Italian legal system lacks a statutory definition of life-sustaining treatments (LST). Law no. 219/2017 does not thoroughly define treatments, nor does it explicitly address artificial nutrition and hydration as forms of LST. Within medical discourse, various interpretations of LST exist, reflecting the intricate nature of modern clinical practices which encompass a comprehensive array of equipment, devices, medications, and medical and nursing interventions tailored to each patient’s unique clinical circumstances. The European Court of Human Rights, in its ruling dated 13 June 2024 [35], referred to “life-sustaining treatments” as interventions designed to achieve legitimate objectives, such as safeguarding the lives of vulnerable individuals who are particularly susceptible to errors and mistreatment, upholding the ethical standards of the medical profession, and preserving the intrinsic value and sanctity of human life. The Constitutional Court dismissed the notion that the existential distress experienced by a terminally ill patient could, in and of itself, compel individual states to legalize medically assisted dying, emphasizing instead the heightened vulnerability of such patients as warranting an approach that inherently incorporates palliative care [22,45,46].

In the Trentini case [47], the Court of Massa [48] had broadly interpreted the requirement of dependence on life-sustaining treatments: this concept not only includes cases of dependence on machines but also covers all medical treatments which, if discontinued, would lead to the patient’s death, even if not immediately, including pharmacological or assistive care. If this approach were followed, all cancer treatments would be life-sustaining treatments. Therefore, if the tumor were irreversible, assisted suicide would be permitted.

The Italian Constitutional Court does not provide a technical–scientific definition of LST but reaffirms that the patient has the right to refuse any medical treatment performed on their body, regardless of how technically complex or invasive it may be [22,49]. The court thus includes LST procedures normally carried out by healthcare personnel who possess specific professional training, but which could also be learned by family members or caregivers who take on the patient’s assistance [22,50]. According to the court, LST should include procedures that are necessary to ensure the performance of vital functions for the patient, so much so that their omission or interruption would foreseeably result in the patient’s death within a short period [22,50,51,52,53,54]. The court provides examples of such procedures: manual bowel evacuation, insertion of urinary catheters, and suction of mucus from the bronchial pathways. When these procedures become necessary to ensure the viability of the patient’s vital functions, to the point that their omission or interruption would predictably lead to death in a short period, they must be considered life-sustaining treatments under the principles established by Judgment no. 242/2019. The patient may lawfully refuse these procedures, just as they can refuse artificial hydration, nutrition, or ventilation, knowing that such a decision will lead to their death within a short time frame [22,55]. Meeting each requirement calls for a high degree of objectivity and clarity, which is essential from a medicolegal perspective, in order to stave off negligence-based malpractice allegations [56,57,58].

The Regional Council of Tuscany, on 11 February 2025, on the occasion of the World Day of the Sick, approved the law “Organizational arrangements for the implementation of the judgments of the Constitutional Court” [59]. This law, consisting of only six articles, stipulates that the application for assisted suicide must be completed within 47 days of submission with the lethal injection of the patient within the region’s health facilities. All costs to complete the process are borne by the region’s health facilities. To that end, the Tuscany Region has phased in funding to the amount of ten thousand euros on a yearly basis for public health facilities for three years (2025, 2026, 2027), to cover expenses (including the purchase of drugs) necessary to allow assisted suicide. The enactment of this law has generated confusion and led to unequal treatment on a regional basis. In fact, it disregards the constitutional principle of equality, which demands equal treatment in the protection of the fundamental right to life throughout the country. However, it is very likely that the Constitutional Court will find it illegitimate because the subject of end-of-life is an exclusive state matter [60].

The paper aims to analyze the requirement of life-sustaining treatments from a legal and comparative perspective in order to evaluate whether the Italian model is preferable to those of Spain, Portugal, and Austria, which are also restrictive.

## 2. Materials and Methods

The authors have drawn upon the Italian scientific databases De Jure and Onelegale, and the international databases PubMed and Scopus, using the following search strings: assisted suicide AND Italy, assisted suicide AND Europe, assisted suicide AND life-sustaining treatments. The authors exclusively considered reviews, research articles, and commentaries published in Italian or English within the past decade in peer-reviewed academic journals. Publications with a sole focus on medical content or those deemed irrelevant to the research objectives were deliberately excluded. After the removal of duplicates, a meticulous selection process based on abstracts was conducted to identify the most pertinent articles, which were then meticulously examined in full. Furthermore, the investigation extended to the relevant works cited in the references of the selected papers. Each publication was scrutinized to discern the rationale behind the evaluations. The authors analyzed the Italian legal system with an analytical method of interpretation of the normative texts and used the comparative method to investigate which of the legal systems considered is preferable.

## 3. Results

The regulations of the abovementioned states have few aspects in common: (a) the patient must be of age, legally capable and competent; (b) their choice to ask for assisted suicide must be autonomous and free from conditioning; (c) the information to be provided must be complete and also include the benefits of palliative care, but the patient remains free to refuse this treatment as well; these are general rules that apply to all health treatments as they are imposed by the necessary respect for the legal and bioethical principle of autonomy; (d) the patient’s consent is always revocable; and (e) the disease must cause intolerable suffering. In Spain, Italy, and Portugal, it is not necessary to measure pain objectively, because suffering can also be merely psychological or intolerable for the patient, and not necessarily unbearable in the traditionally “measurable” sense. In Austria the law is not as clean cut: it holds that suffering cannot be avoided in ways other than assisted suicide. However, given the proven effectiveness of palliative care, if these requirements were interpreted strictly, no patient could be admitted to assisted suicide. Therefore, the words used by the Austrian legislator should also be understood in the same sense as the other regulations considered.

With reference to the other aspects of assisted suicide, the differences are quite important, as succinctly laid in Table 1.

In Spain, Portugal, and Austria, either an acute life-threatening illness or a serious disability can be enough to request assisted suicide.

As for the notion of “acute illness”, it must be incurable and fatal to lawfully warrant assisted suicide in Austria. According to Spanish law, it must entail major alterations in the state of health caused by an accident or illness, unrelated to the patient’s will and marked by constant and intolerable physical or psychological suffering and with a limited prognosis for life, in an overall context of progressive fragility (art. 3, lett. c). Instead, according to Portuguese law, the patient must be suffering from a life-threatening disease, in an advanced and progressive stage, incurable and irreversible, which causes severe suffering (art. 2, letter d). Therefore, with regard to the requirement which entails acute life-threatening conditions, Portuguese law is certainly more restrictive: it in fact combines both the requirement of incurability (provided for by Austrian law) and the advanced stage of disease, akin to the limited life expectancy accounted for by Spanish law.

As for the disability, according to Austrian law, it must be so severe as to permanently impair the patient’s overall quality of life. Portuguese law allows for assisted suicide for patients suffering from serious, permanent, and extensively disabling conditions making them dependent on third parties or on artificial support to carry out the basic activities of daily living, with the certainty or probability that such limitations are permanent, i.e., without the possibility of recovery or any prospect for significant improvement (art. 2, letter e). Spanish law, on the other hand, requires conditions causing the patient limitations that directly affect their physical autonomy, as well as their capacity for expression and social interaction, and associated with constant and intolerable physical or psychological suffering, again with a certainty or high probability that such limitations will never be overcome, with no possibility of recovery or even substantial improvement (art. 3, lett. b). These three legislative frameworks are in fact similar, although the Austrian one appears to be somewhat more generic. The most restrictive seems to be the Spanish one, in that it requires the disability to also impair or severely constrain the patient’s ability to express themselves, whereas the other laws do not account for such an aspect.

In contrast, the Italian Constitutional Court follows a more nuanced trajectory. While it permits assisted suicide solely for patients afflicted with irreversible ailments necessitating life-sustaining interventions, it also extends its scope to encompass a broader spectrum. This inclusive approach considers even non-essential aids, such as manual bowel evacuation, under the umbrella of life-preserving measures. However, this interpretation leads to the classification of laxatives as indispensable medications, a categorization that may appear excessive.

With reference to the procedure for obtaining assisted suicide, the Italian Constitutional Court has charged the public healthcare system with verifying the existence of the requirements for accessing assisted suicide and ensuring the proper execution procedures, which must obviously prevent abuse of vulnerable individuals, guarantee the dignity of the patient, and prevent suffering. The opinion of the locally competent Ethics Committee is also essential (Point 9). As in Italy, also in Spain and Portugal, in order to verify that the patient’s clinical conditions correspond to those required by law, the request for assisted suicide must be evaluated by a specific commission, not only by the team assisting the patient. Therefore, unlike Austria [61], in Italy, Spain, and Portugal, many professionals participate in the decision whether to allow assisted suicide. Furthermore, in Italy, ethics committees include both doctors and lawyers. The same rule is expressly provided for by Portuguese law. The interdisciplinary nature of the commission helps to make a more thoughtful decision.

Regarding the therapeutic relationship, in Italy it is sufficient for a single doctor to evaluate the patient’s conditions and their wishes, and then bring the case to the attention of the ethics committee and the public health facility. Instead, the regulations of other countries require that the case be discussed by two doctors. In Spain and Portugal they must also belong to different teams and one of the two must be a specialist in the pathology from which the patient suffers.

## 4. Discussion

The legal and regulatory frameworks of Spain, Austria, and Portugal share the same basic approach, which emerges from the following principles established by the rulings of the Italian Constitutional Court:

(1) Every life is recognized as carrying inalienable dignity, regardless of the conditions in which it unfolds.

(2) There is no general right to end one’s life, or an absolute right to assisted suicide.

(3) The right to life entails the state’s duty to protect every human life without leaving it insufficiently protected.

(4) The right to refuse any non-legally imposed medical treatment is reaffirmed, even if necessary for survival, regardless of technical complexity or invasiveness.

(5) Rules protecting self-determination must be balanced with the duty to protect human life.

However, beyond the basic similarities and common goals, the rules in force in the abovementioned states are rather different. From a more general perspective, the current Italian regulation certainly appears more generic, due to the lack of an organic, clean-cut legislative framework. The Spanish Portuguese and Austrian laws are therefore more effective in governing key aspects such as the relationship between the patient and the healthcare team, the core features of each request, and the whole procedure, including the period of time that the patient is required to wait before their request is evaluated. Furthermore, in Spain and Portugal the law provides both a preliminary check to verify that the patient is really entitled to assisted suicide and further verification after their death, to ensure there was no abuse.

In Italy, the 2019 Constitutional Court ruling sets the condition of the patient’s clinical conditions, which has to depend upon life-sustaining treatments. This statement can be construed as allowing assisted suicide only for patients with life-threatening disabilities, e.g., vital functions depending on artificial means. Instead, as mentioned earlier on, the 2024 ruling clarified that any irreversible condition that requires treatments whose withdrawal would lead to the patient’s death within a short time, such as manual evacuation of the bowel, is enough to warrant assisted suicide. As a result of the 2024 ruling, the Italian system has significantly come closer to the Spanish, Portuguese, and Austrian approaches. In fact, it allows assisted suicide for people with severely disabling (though not immediately life-threatening) diseases as well.

The difference between these legal systems still rests on terminally ill patients. In Spain, Portugal, and Austria, such patients can accelerate their death by months, although the disease must have reached an advanced stage for such an intervention to be warranted. In the current Italian legal framework, on the other hand, terminally ill patients can resort to assisted suicide only if death occurs in a short time. Still, it is worth noting that no court ruling has unequivocally defined the notion of “short time” yet. However, the cases indicated by the Italian Constitutional Court involve interventions such as aspiration of bronchial mucus and manual evacuation of the intestine, i.e., forms of treatment without which death would occur relatively quickly. That means that said patients would still have to suffer almost throughout their disease as it runs its course, with the sole aid of palliative care.

Three verdicts and one mandate issued by the Constitutional Court were necessary to align Italian regulations more closely with those of Spain, Portugal, and Austria. The Italian journey towards these regulations has been intricate, even convoluted. Through the order of 2018 and the ruling of 2019, the court established highly stringent boundaries due to a case involving a patient dependent on life support for basic functions. However, by 2024, it sanctioned assisted suicide even in cases where the patient’s suffering, though intense, did not reach the same level of severity.

This evolutionary progression, solely reliant on judicial decisions, is limiting as it is contingent on the specifics of individual cases. A comprehensive regulatory framework that accounts for all scenarios and circumstances would necessitate legislative action. However, the Parliament continues to grapple with a reluctance to address ethical dilemmas such as surrogate motherhood and prison conditions. Consequently, despite the court’s proactive stance, the journey towards legislative regularization of assisted suicide appears arduous and protracted.

## 5. Conclusions

From the comparison conducted, none of the abovementioned states appears more permissive in all aspects of the overall regulation.

The Austrian law imposes fewer constraints because neither preventive nor subsequent control by a specific commission is necessary. However, it only allows assisted suicide and not euthanasia. Furthermore, it establishes the longest reflection period.

The Spanish and Portuguese laws, on the one hand, provide for a much more rigorous procedure, but, on the other, also allow euthanasia.

Currently, the Italian system seems to be the most restrictive, because in 2024 the Constitutional Court chose a less broad notion of life-sustaining treatment than that supported by the Court of Massa in the Trentini case. In fact, according to the Constitutional Court, only the patient who would die in a short time without treatment can be helped to die. Conveniently, the other legal systems have not provided for the requirement of being dependent on life-sustaining treatments because it is susceptible to different interpretations and, consequently, can lead to opposite decisions in similar cases.

## Figures and Tables

**Table 1:** Comparison of regulations on assisted suicide (the numbers in parentheses refer to the sections and subsections of the laws).

	Italy	Portugal	Spain	Austria
Regulation currently in force	Constitutional Court rulings no. 242/2019; no. 50/2022; no. 135/2024.	Lei n. 22/2023, passed on 25th May 2023. Regula as condições em que a morte medicamente assistida não é punível e altera o Código Penal [8].	Ley Orgánica 3/2021, de 24 de marzo, de regulación de la eutanasia [6].	Sterbeverfügungsgesetz; Suchtmittelgesetz, Strafgesetzbuch, Änderung. Bundesgesetzblatt I Nr. 242/2021 [10].
Types of end-of-life treatments allowed	Only medically assisted suicide.	Medically assisted suicide. Euthanasia only if the patient is physically unable to self-administer the lethal drug.	Active euthanasia and medically assisted suicide.	Only medically assisted suicide.
Self-determination	Legal capacity; legal age (18 years old); competence; free choice.	Legal capacity; legal age (18 years old); competence; free choice.	Legal capacity; legal age (18 years old); competence; free choice.	Legal capacity; legal age (18 years old); competence; free choice.
Pathological conditions necessary to access assisted suicide	The patient must be (a) suffering from an irreversible disease; (b) and kept alive through life-sustaining treatments.	(1) A life-threatening ailment, in an advanced and progressive stage, incurable and irreversible, causing intense suffering; (2) or harm: (a) severe and devoid of the possibility of remedy or substantial amelioration, and (b) resulting in the individual being reliant on others or technological assistance for carrying out fundamental daily tasks (2).	(1) Limited life expectancy, in a context of progressive frailty; (2) or disabling injury preventing the patient from taking care of themselves in daily life and affecting their ability to express themselves and relate to others, without the possibility of appreciable improvement (3).	(1) An incurable and fatal disease; (2) or a severe long-term illness with persistent symptoms and permanent consequences on the patient’s entire lifestyle (6).
Life-sustaining treatment as a requirement for obtaining assisted suicide	Yes, but interpreted broadly: all treatments without which the patient dies in a short time.	No	No	No
Residence	Not specified	Portuguese citizens or persons legally resident in Portugal (3-2).	Spanish citizens or persons legally resident in Spain (5-1).	Austrian citizens or persons habitually resident in Austria (1-2).
Characteristics of the request for assistance in suicide	A single written request based on an autonomously formed will.	Only one written request, but the patient must sign the document which shows the methods chosen to practice assisted suicide or euthanasia (9-3).	At least two written requests from the patient separated by at least 15 calendar days, unless the physician considers the applicant’s death or loss of his or her capacity to give informed consent to be imminent (5-1(c)).	The dying directive must be drawn up in writing before a notary public or a legally qualified member of the patient representative body after the documentation of the medical information (8-1).
Reflection period	Not specified	At least two months between the assisted suicide request and death (4-5).	Fifteen days between the two requests (5-1(c)).	At least twelve weeks; two weeks only in cases of terminal disease (8).
Therapeutic relationship	The doctor must ensure that the patient is competent and that their choice is free and fully informed, including on the possibility of palliative care.	Same regulation as Spanish law (2, g-h). Doctors and other practitioners involved in the procedure must periodically and frequently listen to the patient’s will (19).	The doctor in charge of the patient’s information and healthcare and a consultant doctor, trained in the field of the pathologies from which the patient suffers and who does not belong to the same team as the previous doctor, must participate in the procedure (3, d-e).	The information must be provided to the patient by two doctors, one of whom is a specialist in palliative medicine (7-1).
Suffering	Physical or psychological suffering that the patient considers unbearable.	Suffering of great intensity, persistent, continuous or permanent and considered intolerable by the person (2).	Constant and intolerable physical or psychological suffering (3).	Suffering that cannot be avoided in any other way (6-3).
Advance directives	No	Not specified	Yes (5-2). Advance directives must be respected by the physician when the patient is incompetent (9-2).	Not specified
Ex-post control (after providing assistance in suicide)	No	Specific Commission (26-2)	Specific commission established in each of the seventeen Spanish regions (18, b).	Not specified
Preventive Control (before providing assistance in suicide)	(1) Public structure of the national health service, and (2) opinion of the local ethics committee.	Specific Commission (8-4)	Specific commission established in each of the seventeen Spanish regions. Assisted suicide may be provided without the aforementioned preventive control in exceptional cases of imminent death or loss of capacity certified by the attending physician (10).	Not specified

## Data Availability

All data herein presented are available upon request from the corresponding author.
